# Free-Water Imaging in White and Gray Matter in Parkinson’s Disease

**DOI:** 10.3390/cells8080839

**Published:** 2019-08-05

**Authors:** Christina Andica, Koji Kamagata, Taku Hatano, Asami Saito, Wataru Uchida, Takashi Ogawa, Haruka Takeshige-Amano, Andrew Zalesky, Akihiko Wada, Michimasa Suzuki, Akifumi Hagiwara, Ryusuke Irie, Masaaki Hori, Kanako K. Kumamaru, Genko Oyama, Yashushi Shimo, Atsushi Umemura, Christos Pantelis, Nobutaka Hattori, Shigeki Aoki

**Affiliations:** 1 Department of Radiology, Juntendo University Graduate School of Medicine, Tokyo 113-8421, Japan; 2 Department of Neurology, Juntendo University School of Medicine, Tokyo 113-8421, Japan; 3 Department of Radiological Sciences, Tokyo Metropolitan University, Graduate School of Human Health Sciences, Tokyo 116-8551, Japan; 4 Melbourne Neuropsychiatry Centre, Department of Psychiatry, The University of Melbourne & Melbourne Health, Parkville, VIC 3053, Australia; 5 Melbourne School of Engineering, The University of Melbourne, VIC 3010, Australia; 6 Department of Radiology, The University of Tokyo Graduate School of Medicine, Tokyo 113-0033, Japan; 7 Department of Radiology, Toho University Omori Medical Center, Tokyo 143-8541, Japan; 8 Department of Neurosurgery, Juntendo University School of Medicine, Tokyo 113-8421, Japan; 9 Florey Institute for Neuroscience and Mental Health, Parkville, VIC 3052, Australia

**Keywords:** Parkinson’s disease, neuronal degeneration, neuroinflammation, diffusion tensor imaging, free-water imaging, tract-based spatial statistics, gray matter-based spatial statistics

## Abstract

This study aimed to discriminate between neuroinflammation and neuronal degeneration in the white matter (WM) and gray matter (GM) of patients with Parkinson’s disease (PD) using free-water (FW) imaging. Analysis using tract-based spatial statistics (TBSS) of 20 patients with PD and 20 healthy individuals revealed changes in FW imaging indices (i.e., reduced FW-corrected fractional anisotropy (FA_T_), increased FW-corrected mean, axial, and radial diffusivities (MD_T_, AD_T_, and RD_T_, respectively) and fractional volume of FW (FW) in somewhat more specific WM areas compared with the changes of DTI indices. The region-of-interest (ROI) analysis further supported these findings, whereby those with PD showed significantly lower FA_T_ and higher MD_T_, AD_T_, and RD_T_ (indices of neuronal degeneration) in anterior WM areas as well as higher FW (index of neuroinflammation) in posterior WM areas compared with the controls. Results of GM-based spatial statistics (GBSS) analysis revealed that patients with PD had significantly higher MD_T_, AD_T_, and FW than the controls, whereas ROI analysis showed significantly increased MD_T_ and FW and a trend toward increased AD_T_ in GM areas, corresponding to Braak stage IV. These findings support the hypothesis that neuroinflammation precedes neuronal degeneration in PD, whereas WM microstructural alterations precede changes in GM.

## 1. Introduction

Parkinson’s disease (PD) is characterized by widespread aggregation of α-synuclein-immunoreactive inclusions in the form of Lewy pathology [1]. Lewy pathology was recently demonstrated to trigger reactive microgliosis before nigral degeneration in animal models of PD [2]. Distinguishing between neuroinflammation and neuronal degeneration in vivo may provide a better understanding of the progression of PD pathology.

Diffusion tensor imaging (DTI) has been widely used in the evaluation of brains of patients with PD [3]. DTI indices such as fractional anisotropy (FA), mean diffusivity (MD), axial diffusivity (AD), and radial diffusivity (RD) characterize the orientation and distribution of the random movements of water molecules, diffusion magnitude, diffusional directionality perpendicular to the axon, and diffusional directionality along the axon, respectively [4]. Despite their sensitivity, DTI indices are not tissue specific [5]. Furthermore, the assumption of a single-tissue compartment per voxel such that partial volume effect averaging in a voxel from free water (FW) [4] can introduce a bias in the interpretation of DTI indices [6]. In the human brain, FW is present as cerebrospinal fluid (CSF); however, it may also accumulate within the extracellular spaces of the brain parenchyma owing to brain pathologies, such as neuroinflammation, tumor, and trauma [7,8]. Studies involving the use of DTI in patients with PD reported reduced FA [9,10] and increased MD [11,12]. A decrease in FA accompanied by increased MD may be attributed to neuronal degeneration and/or neuroinflammation [13]. Thus, the use of these indices might not be useful in differentiating between these pathologies.

Conversely, FW imaging was developed to quantify the contribution of FW and eliminate bias when estimating tissue microstructures and enabled differentiation between alterations in the tissues themselves, such as neuronal degeneration, as measured by FW-corrected DTI indices (FA_T_, MD_T_, AD_T_, and RD_T_, respectively), and extracellular FW changes, such as neuroinflammation, as measured by the fractional volume of FW [7]. FW imaging is achieved by adopting a two-compartment model and fitting 2 tensors into the diffusion data [7]. In PD, FW imaging has thus far only been used to evaluate the substantia nigra. FW within the substantia nigra is considered to be a promising biomarker for distinguishing patients with PD from healthy individuals and as a biomarker for disease progression [14,15].

We hypothesize that applying FW correction to DTI data will improve the detection of abnormalities associated with PD in DTI indices. Specifically, the use of FW imaging might allow the discrimination between neuroinflammation and neuronal degeneration in white matter (WM) and gray matter (GM) in PD. To test this hypothesis, we compared 20 patients with PD to 20 control individuals using DTI and FW imaging.

## 2. Materials and Methods

### 2.1. Subjects

Twenty patients with PD in Hoehn and Yahr stage 1–2 and 20 age- and sex-matched controls with no history of neurologic or psychiatric disorders and no abnormal signals on structural magnetic resonance imaging (MRI) were included in this retrospective case–control study. Patients with PD were diagnosed by specialists based on the clinical diagnostic criteria for PD by the Movement Disorder Society [16]. Disease severity was assessed using non-motor and motor scores of the Movement Disorder Society’s Unified Idiopathic PD Rating Scale (UPDRS) parts I and III, respectively. All patients with PD remained free from atypical parkinsonism and exhibited good response (>30% in UPDRS part III score with change in treatment or a clearly documented history of marked changes from a reliable patient or caregiver [16]) to anti-parkinsonian therapy for 18 months or more after the initial diagnosis. At the time of MRI and clinical examinations, all patients were taking levodopa in combination with a dopamine decarboxylase inhibitor (benserazide or carbidopa). All patients with PD underwent single-photon computed tomography imaging of dopamine transporters and demonstrated deficits in specific binding ratio (less than 95% of the lower limit of prediction intervals for healthy Japanese population) [17]. The clinical phenotypes of PD, including tremor-dominant (*n* = 6), postural instability/gait difficulty (*n* = 8), and intermediate (*n* = 6), were assessed using UPDRS part III in all patients with PD [18]. Furthermore, in all patients with PD, rapid eye movement sleep behavior disorder (RBD) (*n* = 8) was assessed using the RBD single-question screen (RBD1Q) [19]. Table 1 summarizes the demographic and clinical characteristics of healthy controls and patients with PD. The ethics committee approved this study, and all participants signed a written informed consent.

### 2.2. Acquisition of MRI Data

All MRI data were acquired using a 3-T scanner (MAGNETOM Prisma; Siemens Healthcare, Erlangen, Germany) with a 64-channel head coil. Whole-brain diffusion-weighted imaging and 3D magnetization-prepared 180° radio-frequency pulses and rapid gradient-echo (MP-RAGE) T1-weighted imaging were obtained for all subjects. Whole-brain diffusion-weighted images were acquired using spin-echo planar imaging with the following parameters: repetition time, 3300 ms; echo time, 70 ms; flip angle, 90°; diffusion gradient directions, 64; *b*-values, 0 and 1000 s/mm^2^; field of view, 229 × 229 mm; matrix size, 130 × 130; resolution, 1.8 × 1.8 mm; slice thickness, 1.6 mm; and acquisition time, 3 min 55 s. 3D MP-RAGE T1-weighted images were acquired using the following parameters: repetition time, 2300 ms; echo time, 2.32 ms; inversion time, 900 ms; field of view, 240 × 240 mm; matrix size, 256 × 256; resolution, 0.9 × 0.9 mm; slice thickness, 0.9 mm; and acquisition time, 6 min 25 s.

### 2.3. Diffusion MRI Preprocessing

All diffusion MRI data from 64 different axial, sagittal, and coronal directions were visually checked. Moreover, all datasets were free from severe artifacts such as gross geometric distortion, signal dropout, or bulk motion. Diffusion MRI data were then corrected for susceptibility-induced geometric distortions, eddy current distortions, and inter-volume subject motion using EDDY and TOPUP toolboxes [20].

Single-tensor FA, MD, AD, and RD maps were generated using the DTIFIT tool implemented in FMRIB Software Library version 5.0.9 (FSL; Oxford Centre for Functional MRI of the Brain, Oxford, UK; www.fmrib.ox.ac.uk/fsl). Meanwhile, an in-house MATLAB (MathWorks, Natick, MA, USA) script was used to fit a regularized bi-tensor model and generate maps for FA_T_, MD_T_, AD_T_, RD_T_, and FW. A more detailed description of the methods is discussed elsewhere [7].

### 2.4. Voxel-Wise Analysis

Tract-based spatial statistics (TBSS) [21] and GM-based spatial statistics (GBSS) [22] implemented in the FSL [23] were used to regionally map significant differences between groups in all DTI (FA, MD, AD, and RD) and FW imaging (FA_T_, MD_T_, AD_T_, RD_T_, and FW) indices for WM and GM, respectively, as well as to evaluate the relationship of each index with disease duration and clinical scores such as the scores of UPDRS parts I and III.

#### 2.4.1. TBSS

WM was analyzed using the skeleton projection step of TBSS [21] using the following steps. First, FA maps of all subjects were aligned to the standard Montreal Neurological Institute (MNI) space (MNI152) with the FMRIB non-linear registration tool [24]. Next, a mean FA image was generated and thinned to create the mean FA skeleton, which represented the centers of all tracts common to the groups. Next, the threshold of the mean FA skeleton was set to FA > 0.20 to include major WM pathways and exclude peripheral tracts and GM. Finally, the aligned FA map of each subject was projected onto the FA skeleton. The same process was applied to other single-tensor DTI (MD, AD, and RD) and bi-tensor FW imaging (FW, FA_T_, MD_T_, AD_T_, and RD_T_) maps such that the maps were projected onto the mean FA without the initial registration.

#### 2.4.2. GBSS

GM was analyzed using GBSS [22], a GM analog of TBSS, using the following steps. First, the Brain Extraction Tool was used to remove non-brain voxels from each subject’s 3D T1-weighted images. Next, each skull-stripped 3D T1-weighted image was affine- and non-linearly aligned to an MNI152 standard space at a 1-mm resolution using the FMRIB linear image registration tool and the FMRIB non-linear registration tool [24], respectively. Next, field bias was corrected, and GM, WM, and CSF segmentations were obtained using the FMRIB automated segmentation tool [25]. The resulting GM image was then used to create a median GM skeleton with a threshold of 0.2 to minimize the contribution of voxels from WM and CSF. Next, *b*0 maps of each subject were affine-aligned to their 3D T1-weighted images (epi-reg). After all maps were affine- and non-linearly aligned into an MNI152 brain common space at a 1-mm resolution [24], the aligned maps of each subject were projected onto the median GM skeleton map.

### 2.5. Region-of-Interest Analysis

WM and GM were further evaluated using automatic region-of-interest (ROI) analyses. Maps showing significant clusters on TBSS and GBSS analyses were localized using Johns Hopkins University’s ICBM-DTI-81 WM labels and tractography atlases and the Desikan–Killiany atlas, respectively. The average diffusion metric was averaged over all WM and GM skeleton voxels comprising a given region delineated by the atlases for all subjects.

Based on the TBSS results, WM was divided into an anterior portion, comprising forceps minor, bilateral anterior corona radiata, and anterior thalamic radiation, and a posterior portion comprising forceps major, bilateral posterior corona radiata, and posterior thalamic radiation.

PD progresses according to the distribution pattern of Lewy pathology deposition defined by Braak staging, whereas motor symptoms appear when the Lewy pathology reaches stage III [26]. Given that all patients with PD included in the present study showed motor symptoms at the time of examination, GM was analyzed based on Braak staging, which comprised hippocampus, para-hippocampal gyrus, amygdala, cingulate cortices, and thalamus for stage IV; temporal, frontal and parietal association cortices and striatum for stage V; and primary sensory or motor cortices for stage VI [27,28].

### 2.6. Voxel-Based Morphometry

Voxel-based morphometry was used to obtain WM and GM volumetry. First, 3D T1-weighted images were segmented into GM, WM, and CSF through a unified tissue segmentation model using the Statistical Parametric Mapping 12 software (Wellcome Department of Imaging Neuroscience, London, UK; http://www.fil.ion.ucl.ac.uk/spm/software/spm12/) running on a MATLAB 2014a platform (MathWorks; https://www.mathworks.com/products/matlab.html) [29]. Segmented GM and WM images were then spatially normalized to the customized template in the standardized anatomic space using the Diffeomorphic Anatomical Registration Through Exponentiated Lie Algebra (DARTEL) algorithm [30]. To preserve GM and WM volumes within each voxel, Jacobean determinants derived from spatial normalization using DARTEL and an 8 mm full-width at half maximum Gaussian kernel were used to modulate and smooth the images, respectively.

### 2.7. Statistical Analysis

All statistical analyses were performed using IBM SPSS Statistics for Windows, version 22.0 (IBM Corporation, Armonk, NY, USA), except for general linear model analysis, where the FSL [23] was used. The Shapiro–Wilk test was used to assess data normality, whereas demographic data were analyzed using unpaired Student’s *t*-test and the χ^2^ test for continuous and categorical variables, respectively. Statistical significance for all two-tailed tests was set at 0.05.

For TBSS and GBSS analyses, a general linear model framework including unpaired Student’s *t*-test (healthy controls vs. all patients with PD) and one-way analysis of variance (healthy controls vs. patients with right-sided PD vs. those with left-sided PD), with age and sex as covariates, and the randomize tool was used with 5000 permutations to compare all diffusion indices between groups. Results were then corrected for multiple comparisons by controlling for family wise error (FWE) and applying threshold-free cluster enhancement. An FWE-corrected *P* value of 0.05 was considered to indicate statistical significance. The randomize tool was also applied during voxel-wise correlation analysis of each index with disease duration, UPDRS part I score, UPRDRS part I subscores (cognitive, neuropsychiatric, sleep disorder, sensory and others, and autonomic), or UPDRS part III score.

Unpaired Student’s *t*-test was used to assess differences between the healthy control and PD groups according to the ROI of each metric that showed significant group differences in the TBSS analysis of WM and GM. Bonferroni’s correction was used for multiple comparisons in WM (*n* = 2, anterior and posterior) and GM (*n* = 3, stages IV, V, and VI) with the level of significance for two-tailed *P* values set at 0.025 (0.05/2) and 0.017 (0.05/3), respectively. The effect size was then calculated using Cohen’s *d* [31] to evaluate the statistical power of the relationship determined during group comparisons. Average diffusion metrics of the WM and GM ROIs were then correlated with disease duration or UPDRS part I score, UPDRS part I subscores (cognitive, neuropsychiatric, sleep disorder, sensory and others, and autonomic), or UPDRS part III score. Considering the exploratory nature of this analysis, Bonferroni correction was not applied.

The volumes were compared between the patients with PD and healthy controls using a generalized linear model for analysis of covariance, with age, sex, and total intracranial volume as covariates using the FWE rate set at *p* = 0.05.

## 3. Results

### 3.1. WM Alterations

#### 3.1.1. TBSS

**Figure 1:**
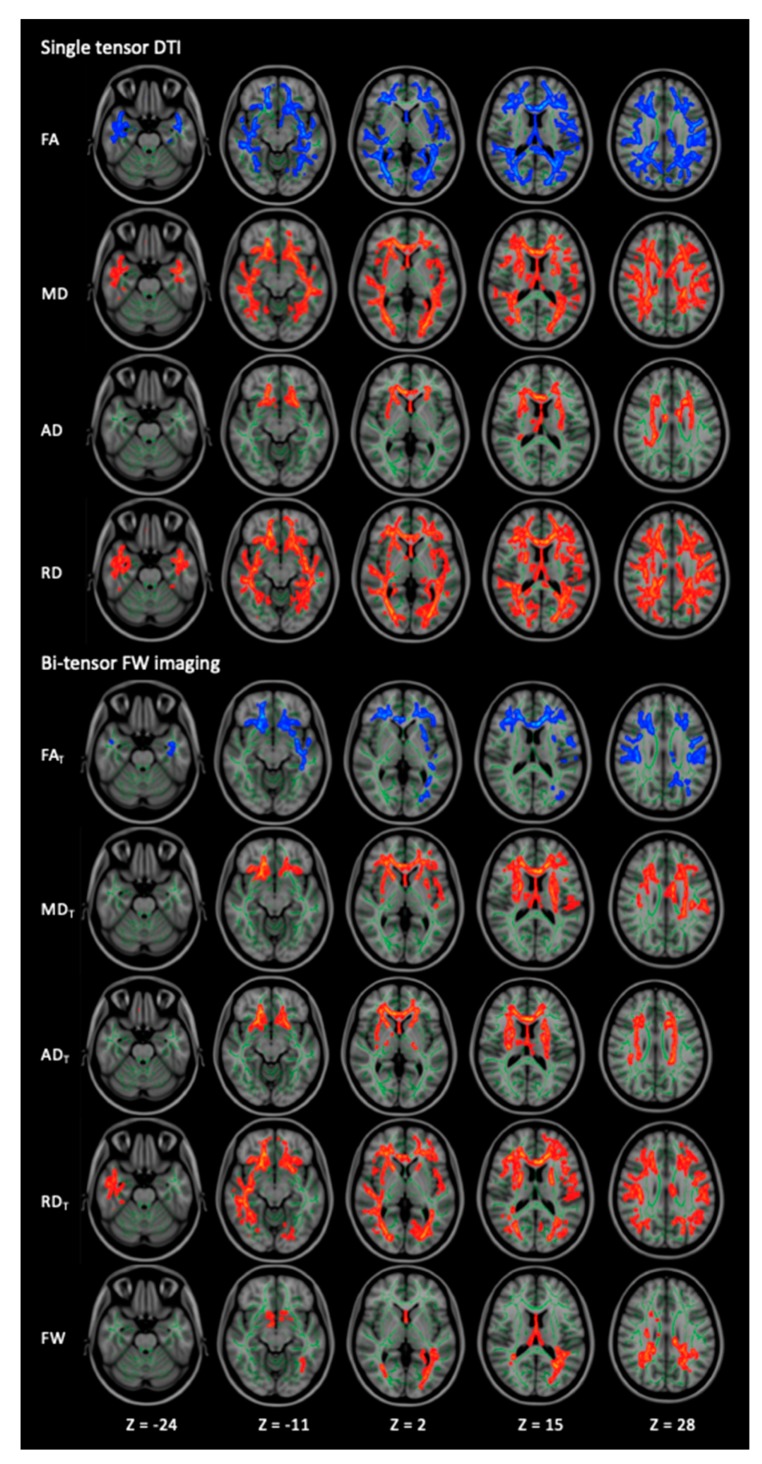
Comparison of DTI (FA, MD, AD, and RD) and FW imaging (FA_T_, MD_T_, AD_T_, RD_T_, and FW) indices between healthy controls and patients with PD. TBSS analyses show that patients with PD have significantly (*p* < 0.05, FWE-corrected) lower FA and FA_T_ (blue/light-blue voxels) and significantly higher MD, MD_T_, AD, AD_T_, RD, RD_T_, and FW (red-yellow voxels) compared with healthy controls. The skeleton is presented in green. To aid visualization, the results are thickened using the fill script implemented in the FMRIB Software Library. AD, axial diffusivity; AD_T_, free water-corrected axial diffusivity; DTI: diffusion tensor imaging; FA, fractional anisotropy; FA_T_, free water-corrected fractional anisotropy; FW, free water; MD, mean diffusivity; MD_T_, free water-corrected mean diffusivity; PD, Parkinson’s disease; RD, radial diffusivity; RD_T_, free water-corrected radial diffusivity; TBSS, tract-based spatial statistics.

Figure 1 and Table 2 show the results of the TBSS analysis for DTI (FA, MD, AD, and RD) and FW (FA_T_, MD_T_, AD_T_, RD_T_, and FW) imaging indices. The patients with PD exhibited significantly lower FA and FA_T_ and higher MD, MD_T_, AD, AD_T_, RD, RD_T_, and FW (*p* < 0.05, FWE-corrected) compared with the healthy controls. While changes in the DTI indices were observed across broad areas, changes in the FW imaging indices were observed in relatively limited areas. Furthermore, the reduced FA_T_ and the increased MD_T_ and AD_T_ were predominantly observed in the anterior portion, whereas the increased FW was observed in the posterior WM. The details on the anatomical region, peak *t*-value, and peak MNI coordinates of significant clusters are presented in Table 2. There were no significant differences in the DTI and FW imaging indices between the patients with right-sided and left-sided onset PD, the healthy controls and the patients with right-sided onset PD, or the healthy controls and the patients with left-sided onset PD.

#### 3.1.2. ROI

**Figure 2:**
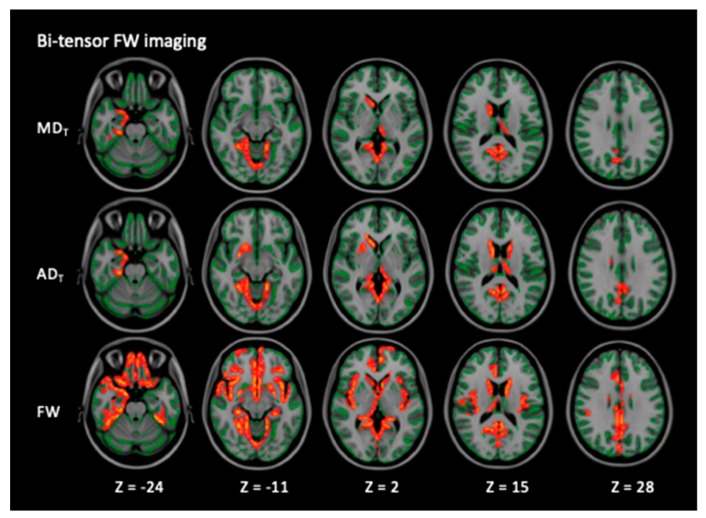
Comparison of FW imaging (MD_T_, AD_T_, and FW) indices between the healthy controls and the patients with PD. GBSS analyses showed that the patients with PD had significantly higher MD_T_, AD_T_, and FW (red-yellow voxels) compared with the healthy controls (*p* < 0.05, FWE-corrected). The skeleton is presented in green. To aid visualization, the results are thickened using the fill script implemented in the FMRIB Software Library. AD, axial diffusivity; AD_T_, FW-corrected axial diffusivity; FW, free water; GBSS, gray matter-based spatial statistics; MD, mean diffusivity; MD_T_, FW-corrected mean diffusivity; PD, Parkinson’s disease.

Figure 2 and Table 3 show the ROI analysis results for DTI (FA, MD, AD, and RD) and FW imaging (FA_T_, MD_T_, AD_T_, RD_T_, and FW) indices in the anterior and posterior WM tracts. Within the anterior WM tracts, the patients with PD exhibited significantly lower FA and FA_T_ and higher MD, RD, MD_T_, AD_T_, and RD_T_ compared with the healthy controls. Moreover, the patients with PD tended to have higher AD compared with the healthy controls. Within the posterior WM tracts, the patients with PD exhibited significantly lower FA and higher RD and FW compared with the healthy controls. Moreover, the patients with PD tended to have higher MD compared with the healthy controls.

### 3.2. GM Alterations

#### 3.2.1. GBSS

**Figure 3:**
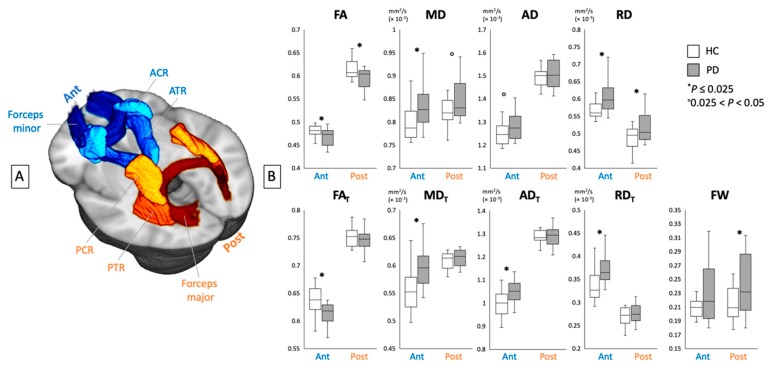
(**A**) Region-of-interest analyses of the anterior (ACR, ATR, and forceps minor) and posterior (PCR, PTR, and forceps major) white matter areas. (**B**) Mean values for DTI (FA, MD, AD, and RD) and FW imaging (FA_T_, MD_T_, AD_T_, RD_T_, and FW) indices of the anterior and posterior white matter areas in healthy controls (white bars) and patients with PD (gray bars). ACR, anterior corona radiata; AD, axial diffusivity, AD_T_, FW-corrected axial diffusivity; ATR, anterior thalamic radiation; DTI, diffusion tensor imaging; FA, fractional anisotropy; FA_T_, free water-corrected fractional anisotropy; FW, free water; HC, healthy controls; MD, mean diffusivity; MD_T_, free water-corrected mean diffusivity; PCR, posterior corona radiata; PD, Parkinson’s disease; PTR, posterior thalamic radiation; RD, radial diffusivity; RD_T_, free water-corrected radial diffusivity.

Figure 3 and Table 4 show the GBSS analysis results of MD_T_, AD_T_, and FW. The patients with PD exhibited significantly higher MD_T_, AD_T_, and FW (*p* < 0.05, FWE-corrected) indices compared with the healthy controls. The details regarding the anatomical region, peak *t*-value, and peak MNI coordinates of significant clusters are presented in Table 4. No significant differences in any of the DTI indices, FA_T_, or RD_T_ were found between the patients with PD and the healthy controls. There were no significant differences in the DTI and FW imaging indices between the patients with right-sided and left-sided onset PD, the healthy controls and the patients with right-sided onset PD, or the healthy controls and the patients with left-sided onset PD.

#### 3.2.2. ROI

**Figure 4:**
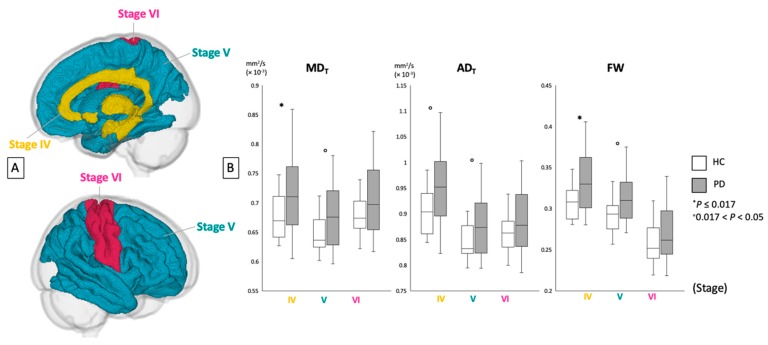
(**A**) Region-of-interest analyses of gray matter areas belonging to Braak stages IV, V, and VI. (**B**) Mean values for FW imaging indices (MD_T_, AD_T,_ and FW) of each area in healthy controls (white bars) and patients with PD (gray bars). AD_T_, free water-corrected axial diffusivity; FW, free water; HC, healthy controls; MD_T_, free water-corrected mean diffusivity; PD, Parkinson’s disease.

Figure 4 and Table 5 show the ROI analysis results for MD_T_, AD_T_, and FW in GM areas corresponding to Braak stages IV–VI. The patients with PD demonstrated significantly higher MD_T_ and FW and tended to have higher AD_T_ compared with the healthy controls in the areas corresponding to Braak stage IV. Among the Braak stage V areas, the patients with PD tended to have higher MD_T_, AD_T_, and FW compared with the healthy controls. No significant differences in GM were found between the patients with PD and healthy controls within the areas corresponding to Braak stage VI.

### 3.3. WM and GM Volumetry

Morphometry analysis revealed no focal volumetric differences between the patients with PD and the healthy controls.

### 3.4. Correlation Analysis

Both the voxel-wise and the ROI analyses showed no significant correlations between the indices and the disease duration or clinical scores measured using UPDRS part I score, UPDRS part I subscores, and UPDRS part III score.

## 4. Discussion

In the present study, we investigated WM and GM alterations by comparing the patients with PD to the healthy controls using DTI and FW images. In the TBSS analysis, the changes in the FW imaging indices (increased FW and reduced FA_T_ and increased MD_T_, AD_T_, and RD_T_) were in somewhat more specific WM areas compared with the changes in DTI indices (reduced FA and increased MD, AD, and RD). These findings are further supported by our ROI analysis to specifically analyze the WM tracts running through the anterior and posterior portions of the brain. The significantly reduced FA_T_ and the significantly increased MD_T_, AD_T_, and RD_T_ were observed only in the anterior WM tracts with a higher effect size than the DTI indices; the increased FW was observed only in the posterior WM tracts of the patients with PD. Furthermore, in the GBSS analysis, increases in MD_T_, AD_T_, and FW were observed in the GM of the patients with PD, whereas no significant differences were found in any of the DTI indices. The ROI analysis demonstrated that the changes in these measures corresponded with Braak stage IV.

In the WM, the observed increase in increased FW, which is expected during neuroinflammation [32], is in line with the reported inflammatory processes related to the activation of astrocytes and microglia, which are linked to α-synuclein aggregation, in PD [33]. Furthermore, the reduced FA_T_ and increased MD_T_, AD_T_, and RD_T_, which usually result from axonal damage and demyelination [4], might be the result of the aggregation of Lewy neurites that are associated with impaired axonal transport with subsequent microstructural changes in the axon and surrounding myelin [11]. The current study also demonstrated that the FW indices were able to derive more precise estimations of localized WM degeneration in PD compared with the DTI indices. An explanation might be that partialling out FW eliminated the influence of CSF on WM tracts running closely adjacent to the ventricles and brain pathologies such as neuroinflammation, thereby increasing the specificity of FW imaging indices [7,8]. Thus, the current results suggest that the changes in the DTI indices within the posterior WM tracts of the patients with PD were largely influenced by neuroinflammation. In line with the current results, a study on patients with schizophrenia reported changes in DTI indices in specific areas after excluding the influence of extracellular FW [8]. Furthermore, a study recently demonstrated the correlation between FW obtained using FW imaging and the 18-kDa translocator protein (TSPO), using positron emission tomography with [^11^C]DPA-713 that binds TSPO, in patients with traumatic brain injury [34].

Anterior brain has been suggested to be more prone to Lewy pathology than posterior brain [35,36]. The prefrontal cortex is an area where Lewy pathology occurs at a relatively early stage of PD [11]. Additionally, PD is considered to be a prion-like disorder where misfolded α-synuclein spreads from one neuron to another from the anterior to the posterior along WM [26]. Given that most of the current study patients (85%) were in Hoehn and Yahr stage 2, it is possible that, at the time of the examination, the Lewy neurites had progressed from the anterior portions to reach the posterior portions of the brain, with simultaneous neurodegeneration induced by neuroinflammation in the anterior portions. Taken together, the current results demonstrated that the microstructural changes in the WM of patients with PD were preceded by neuroinflammation and followed by neurodegeneration. These findings are supported by the previously demonstrated exacerbation of dopaminergic neurodegeneration in the substantia nigra pars compacta by the chronic release of pro-inflammatory cytokines [33]. Moreover, animal models of PD demonstrated that Lewy pathology triggered reactive microgliosis prior to nigral degeneration [2].

Increased MD_T_, AD_T_, and FW, which are indicative of axonal degeneration and neuroinflammation [4,32], in the GM of patients with PD, were demonstrated using GBSS analysis. The lack of significant differences in the DTI indices in GM suggests that the FW imaging indices provided greater sensitivity in the detection of GM abnormalities in the patients with PD. DTI is not the preferred method for the evaluation of GM, especially the cortex, other than the substantia nigra and striatum, mainly because of its inability to thoroughly describe microstructural abnormalities in GM due to water diffusion isotropy [37]. Furthermore, the use of diffusion MRI for the evaluation of GM, especially cortical areas, has been limited by the partial volume effect of the WM and CSF adjacent to the cortical GM [38]. FW imaging removes the isotropic FW compartment in the GM, thus leading to a more anisotropic estimation of the single tensor in one voxel [39]. In the present study, the partial volume effect of the WM and CSF was further minimized using GBSS analysis. In line with a previous study [28], the current results demonstrated that GBSS analysis enables the regional characterization of GM pathology in PD because GBSS analysis involves the aggregation of diffusion MRI indices in regions surrounding the skeleton created at the center of the cortical GM, thus minimizing the partial volume effect [22].

Unlike the widespread microstructural changes observed in WM, the GBSS results identified significant changes within more limited GM areas in the patients with PD. Furthermore, no significant difference in FA_T_ of the GM was observed between the patients with PD and the healthy controls. Decreases in GM FA, an index of neuron integrity, was previously reported to appear later than increases in MD during the progression of PD [40]. Taken together, the current results indicate that WM microstructural changes in PD occur early and may precede changes in GM. Our findings agree with those of previous studies that reported widespread changes in WM microstructure in patients with early PD with no or limited GM alteration [41,42,43]. These results are also consistent with a recent hypothesis regarding pathological progression in PD, wherein pre-synaptic terminal damage, impaired axonal transport, and/or altered axonal structure precede cell body damage, otherwise known as the “dying back” pattern of degeneration [44]. In addition, the changes in FW imaging measures were also demonstrated in the thalamus areas. Previous histopathological and imaging studies demonstrated the involvement of thalamus in PD patients with RBD [45,46]. Thus, the microstructural changes of the thalamus might be related to RBD in patients with PD.

Furthermore, the ROI analyses in the present study indicated that the most striking difference in the extent and the distribution of GM damage, specifically, neuroinflammation (indexed by FW) and axonal degeneration (indexed by MD_T_ and AD_T_) [4,32], corresponded with Braak stage IV. At Braak stage III, patients with PD exhibit typical features described in Hoehn and Yahr stage 1 (i.e., tremors, rigidity, and bradykinesia) typically on one side of the body [47]. However, as the disease enters Braak stage IV, the clinical features become bilateral (Hoehn and Yahr stage 2). Considering that 85% of the participants in the present study were in Hoehn and Yahr stage 2, the study findings agree with the neuropathological and clinical patterns of PD. Additionally, among the changes in MD_T_, AD_T_, and FW, FW showed the strongest effect size. This finding, along with that for WM, also suggest that neuroinflammation may be preceding axonal degeneration in the GM of patients with PD.

No differences in the WM and GM volumes were observed between the patients with PD and healthy controls. The current study results support the conclusions from several studies wherein no volume changes were detected in early PD [28,42], suggesting that volumetric alterations in brain structures may occur later in the disease course, as described in studies on advanced PD [41]. This finding also lends further support for the suggestion that diffusion MRI indices are potentially more sensitive as biomarkers for detecting microstructural changes in PD prior to tissue loss detected during volumetric MRI.

None of the indices included herein correlated with disease duration. This finding is consistent with those of histopathological studies showing that the aggregation of Lewy pathology occurs before the appearance of symptoms [48]. However, medication effects may also explain the observed lack of correlation with the scores of UPDRS parts I and III [49]. Zhang et al. [50] and Wen et al. [51] previously reported a correlation between DTI indices and the scores of UPDRS part III in the brains of untreated patients with PD. In contrast, we [52] and another group [49] showed a lack of such correlation in the brains of treated patients with PD. Furthermore, one study demonstrated that the UPDRS lacks the sensitivity to discriminate the severity of symptoms of early PD [53].

Some limitations of the present study should be noted. First, the small sample size limits the statistical power, which may have led to the nonsignificant results in some measures including correlation analysis. Second, this was a case–control study. Third, although the patients with PD in the current study were defined to be Hoehn and Yahr stage 1–2, most of the patients were in Hoehn and Yahr stage 2. Further studies including patients with preclinical or earlier-stage PD and longitudinal studies involving larger cohorts will be particularly informative in clarifying the utility of FW indices as biomarkers for diagnosis and disease progression and in resolving the temporal sequence of events of neuroinflammation and neurodegeneration. Fourth, the current study lacked histopathological verification. Future studies should also include TSPO-positron emission tomography imaging to verify neuroinflammation in PD. Fifth, in the current study, we did not categorize the patients with PD based on the clinical PD phenotypes such as non-tremor and tremor-dominant subtypes, whereas motor phenotypes in PD are recognized to have a distinct neural basis [54], which should be considered for further investigation as well. Sixth, we did not specifically evaluate patients with PD based on clinical symptoms such as RBD or hyposmia. Additionally, polysomnography [55] and the odor stick identification test [56], objective assessments that confirm the clinical diagnosis of RBD and hyposmia, respectively, were not performed in the patients with PD. However, considering that both symptoms appear early and are common in PD, further studies should be conducted with objective evaluation focusing on these symptoms. Studies investigating microstructural changes in patients with RBD, de novo PD, and PD with RBD, using FW imaging will also be crucial for a better understanding of the pathology underlying PD. Finally, TBSS and GBSS lack the sensitivity to detect peripheral effects located outside the skeleton, and the skeleton projection step may also introduce bias to the projected parameters.

## 5. Conclusions

The current study results provide novel evidence that FW imaging in PD may be useful in determining the etiology of microstructural changes, more specifically related to neurodegeneration and neuroinflammation. Furthermore, FW imaging provided more precise estimations of localized microstructural changes in PD compared with DTI. These findings also demonstrated that neuroinflammation preceded neurodegeneration in PD, whereas changes in the WM microstructure preceded those in the GM. However, considering the limitations of the current study, the findings should be clinically interpreted with caution. Particularly, longitudinal studies and histopathological verification are necessary to validate the current study findings.

## Figures and Tables

**Table 1:** Demographic characteristics of the study participants.

	HC	All PD	Right-Sided Onset PD	Left-Sided Onset PD	*P* Value (HC vs. All PD	*P* Value (R vs. L Onset PD)	*P* Value (HC vs. R Onset PD)	*P* Value (HC vs. L Onset PD)
Number	20	20	12	8	—	—	—	—
Sex, *N* (male/female) °	12/8	11/9	5/7	6/2	0.75	0.14	0.31	0.45
Age (mean years ± SD) *	67.15 ± 1.18	65.05 ± 10.9	63.50 ± 11.13	67.38 ± 8.43	0.52	0.16	0.15	0.91
Disease duration (mean years ± SD)	—	6.95 ± 3.93	7.5 ± 3.63	6.13 ± 4.45	—	0.46	—	—
MDS-UPDRS part I (mean ± SD) *	—	5.45 ± 2.93	5.25 ± 2.45	5.75 ± 3.69	—	0.72	—	—
MDS-UPDRS part I subscores (mean ± SD) *								
Cognitive (I.1)	—	0.15 ± 0.37	0.17 ± 0.39	0.13 ± 0.35	—	0.81	—	—
Neuropsychiatric (I.2–I.6)	—	1.20 ± 1.32	1.00 ± 1.04	1.50 ± 1.69	—	0.42	—	—
Sleep disorder (I.7, I.8)	—	1.45 ± 1.28	1.25 ± 1.22	1.75 ± 1.39	—	0.41	—	—
Sensory and others (I.9, I.13)	—	0.90 ± 1.02	1.83 ± 1.11	1.63 ± 0.74	—	0.61	—	—
Autonomic (I.10–I.12)	—	1.75 ± 0.97	1.83 ± 1.11	1.63 ± 0.74	—	0.65	—	—
MDS-UPDRS part III (mean ± SD) *	—	11.05 ± 5.22	11.25 ± 4.86	10.75 ± 6.04	—	0.84	—	—
Hoehn and Yahr staging (mean ± SD) *	—	1.85 ± 0.37	1.75 0.45	2 ± 0		0.14	—	—
1, *N* (%)	—	3 (15%)	3 (25%)	0 (0%)	—	—	—	—
2, *N* (%)	—	17 (85%)	9 (75%)	8 (100%)	—	—	—	—
LED (mean ± SD) *	—	862.25 ± 596.50	898.75 ± 607.84	807.50 ± 616.02	—	0.75	—	—
Mean SBR (mean ± SD)	—	3.28 ± 1.27	—	—	—	—	—	

HC, healthy controls; LED, levodopa equivalent dose; L, left; MDS-UPDRS, Movement Disorder Society’s Unified Parkinson’s Disease Rating Scale; PD, Parkinson’s disease; R, right; SBR, specific binding ratio; SD, standard deviation. *Note*. Statistical analyses were performed using the χ^2^ test (°) or unpaired Student’s *t*-test (*). MDS-UPDRS part I subscores: (1) cognitive impairment, (2) hallucinations, (3) depression, (4) anxiety, (5) apathy, (6) dopamine dysregulation syndrome, (7) sleep problems, (8) daytime sleepiness, (9) pain, (10) urinary problem, (11) constipation, (12) light headedness on standing, (13) fatigue.

**Table 2:** Tract-based spatial statistics analysis of diffusion tensor and free-water imaging indices in patients with Parkinson’s disease and healthy controls.

Modality	Contrast	Cluster Size	Anatomical Region	Peak *t*-Value	Peak MNI Coordinates (*X*, *Y*, *Z*)
Single-tensor DTI					
FA	HC > PD	46483	Bilateral ATR, CST, CgH, IFOF, ILF, SLF, UF, temporal part of the SLF, retrolenticular part of the IC, ACR, SCR, PCR, PTR, sagittal stratum, external capsule, tapatum; Lt-CCG; Rt-PLIC; fornix, forceps major and minor, genu, body and splenium of CC	6.81	133, 124, 44
MD	HC < PD	39448	Bilateral ATR, CST, IFOF, ILF, SLF, UF, temporal part of the SLF, ALIC, PLIC, retrolenticular part of the IC, ACR, SCR, PCR, PTR, sagittal stratum, external capsule, SFOF, tapatum; genu, body and splenium of CC, fornix and forceps major and minor	5.9	113, 160, 76
AD	HC < PD	8520	Bilateral ATR, CST, IFOF, UF, ALIC, ACR, SCR, external capsule, SFOF; Lt-PLIC; Rt-SLF, PCR, retrolenticular part of the IC, fornix; genu and body of CC, forceps minor	5.49	113, 160, 76
RD	HC < PD	54131	Bilateral ATR, CST, CgH, IFOF, ILF, SLF, UF, temporal part of the SLF, medial lemniscus, ICP, SCP, ALIC, PLIC, retrolenticular part of the IC, ACR, SCR, PCR, PTR, sagittal stratum, external capsule, SFOF, tapatum; Lt-CCG; genu, body and splenium of CC, fornix, forceps major and minor and MCP	5.95	121, 106, 64
Bi-tensor FW imaging					
FA_T_	HC > PD	22185	Bilateral ATR, CST, IFOF, ILF, SLF, ACR, SCR, PCR, external capsule; Lt-CCG, UF, retrolenticular part of the IC, PTR, sagittal stratum; forceps major and minor, genu, body and splenium of CC, fornix	5.37	45, 125, 47
MD_T_	HC < PD	18356	Bilateral ATR, CST, IFOF, SLF, UF, ALIC, PLIC, ACR, SCR, external capsule, SFOF; Lt-CCG, retrolenticular part of the IC, PCR; forceps minor, genu, body and splenium of CC; fornix	5.73	119, 94, 120
AD_T_	HC < PD	11610	Bilateral ATR, CST, IFOF, UF, ALIC, PLIC, retrolenticular part of the IC, ACR, SCR, PCR, external capsule, SFOF; Rt- SLF; genu, body and splenium of the CC and forceps minor	5.52	80, 158, 77
RD_T_	HC < PD	33504	Bilateral ATR, CST, IFOF, ILF, SLF, UF, ALIC, PLIC, ACR, SCR, PCR, PTR, sagittal stratum, external capsule, SFOF; Lt-CCG; temporal part of the Rt-SLF, retrolenticular part of the IC, UF, tapatum; forceps major and minor, genu, body and splenium of CC, fornix	5.64	143, 99, 99
FW	HC < PD	5716	Bilateral ATR, CST, IFOF, ILF, SLF, SLF temporal part, SCR, PCR, PTR, tapatum; Lt-retrolenticular part of the IC, sagittal stratum; Rt-ACR; forceps major and minor, genu, body and splenium of CC, fornix	5.45	89, 133, 74

Lt, left; Rt, right; ACR, anterior corona radiata; AD, axial diffusivity; AD_T_, free water-corrected axial diffusivity; ALIC, anterior limb of the internal capsule; ATR, anterior thalamic radiation; CC, corpus callosum, CCG, cingulum cingulate gyrus; CgH, cingulum hippocampus; CST, corticospinal tract; DTI, diffusion tensor imaging; FA, fractional anisotropy; FA_T_, free water-corrected fractional anisotropy; FW, free water; HC, healthy control; IC, internal capsule; IFOF, inferior fronto-occipital fasciculus; ILF, inferior longitudinal fasciculus; MD, mean diffusivity; MD_T_, free water-corrected mean diffusivity; MNI, Montreal Neurological Institute; PCR, posterior corona radiata; PD, Parkinson’s disease; PLIC, posterior limb of the internal capsule; PTR, posterior thalamic radiation; RD, radial diffusivity; RD_T_, free water-corrected radial diffusivity; SCR, superior corona radiata; SFOF, superior fronto-occipital fasciculus; SLF, superior longitudinal fasciculus; UF, uncinate fasciculus.

**Table 3:** Region-of-interest analysis of diffusion tensor and free-water imaging indices in the white matter of Parkinson’s disease patients and healthy controls.

	WM Areas	HC		PD		*P* Value	*t*-Value	Cohen’s *d*
		Mean	SD	Mean	SD			
**DTI**								
**FA**	Anterior	0.48	0.014	0.47	0.018	0.019 *	2.46	0.78
	Posterior	0.61	0.021	0.60	0.021	0.011 *	2.69	0.85
**MD**	Anterior	0.80	0.035	0.83	0.048	0.014 *	−2.58	0.81
	Posterior	0.82	0.029	0.85	0.047	0.036 °	−2.18	0.69
**AD**	Anterior	1.25	0.043	1.28	0.058	0.038 °	−2.16	0.68
	Posterior	1.49	0.038	1.51	0.058	0.32	−1.00	0.32
**RD**	Anterior	0.57	0.032	0.61	0.046	0.010 *	−2.71	0.86
	Posterior	0.49	0.032	0.52	0.045	0.012 *	−2.65	0.84
**FW imaging**								
**FA_T_**	Anterior	0.64	0.026	0.61	0.020	0.0021 *	3.30	1.04
	Posterior	0.75	0.018	0.75	0.019	0.40	0.85	0.27
**MD_T_**	Anterior	0.56	0.039	0.60	0.036	0.0014 *	−3.45	1.09
	Posterior	0.61	0.014	0.62	0.022	0.25	−1.16	0.37
**AD_T_**	Anterior	1.00	0.053	1.05	0.053	0.0025 *	−3.24	1.02
	Posterior	1.29	0.027	1.29	0.041	0.71	−0.38	0.12
**RD_T_**	Anterior	0.34	0.033	0.37	0.031	0.0016 *	−3.40	1.22
	Posterior	0.27	0.020	0.28	0.024	0.26	−1.14	0.36
**FW**	Anterior	0.21	0.016	0.23	0.042	0.079	−1.81	0.57
	Posterior	0.21	0.024	0.24	0.042	0.020 *	−2.44	0.77

AD, axial diffusivity; AD_T_, free water-corrected axial diffusivity; DTI, diffusion tensor imaging; FA, fractional anisotropy; FA_T_, free water-corrected fractional anisotropy; FW, free water; HC, healthy control; MD, mean diffusivity; MD_T_, free water-corrected mean diffusivity; PD, Parkinson’s disease; RD, radial diffusivity; RD_T_, free water-corrected radial diffusivity; SD, standard deviation; WM, white matter. * *p* ≤ 0.025; ° 0.025 < *p* < 0.05.

**Table 4:** Gray-matter-based spatial statistics analysis of free-water imaging indices in patients with Parkinson’s disease and healthy controls.

Modality	Contrast	Cluster Size	Anatomical Region		Peak *t*-Value	Peak MNI Coordinates (*X*, *Y*, *Z*)
MD_T_	HC < PD				4.9	38, 60, 46
		—	Frontal	—		
		42	Temporal	Bilateral fusiform, Rt-entorhinal and temporal pole		
		43	Parietal	Bilateral precuneus		
		23	Occipital	Bilateral lingual		
		102	Limbic and para-limbic	Bilateral isthmus cingulate and para-hippocampal; Rt-hippocampus		
		49	Deep GM	Lt-thalamus; Rt-caudate and putamen		
AD_T_	HC < PD				5.05	38, 60, 46
		—	Frontal	—		
		62	Temporal	Bilateral fusiform; Rt-enthorinal, inferior temporal and temporal pole		
		53	Parietal	Bilateral precuneus		
		27	Occipital	Bilateral lingual		
		146	Limbic and para-limbic	Bilateral isthmus cingulate, para-hippocampal and hippocampus; Rt-amygdala and accumbens		
		162	Deep GM	Bilateral thalamus, caudate; Rt-putamen		
FW	HC < PD				7.37	43, 46, 54
		534	Frontal	Bilateral lateral orbitofrontal, medial orbitofrontal, pars opercularis, pars orbitalis, pars triangularis, superior frontal, frontal pole and precentral; Rt-paracentral and rostral middle frontal		
		235	Temporal	Bilateral fusiform and superior temporal; Lt-transverse temporal; Rt-entorhinal, fusiform, inferior temporal and temporal pole		
		132	Parietal	Bilateral post-central, precuneus; Rt-supramarginal		
		47	Occipital	Bilateral lingual; Lt-cuneus, lateral-occipital and pericalcarine		
		1106	Limbic and para-limbic	Bilateral isthmus cingulate, caudal anterior cingulate, posterior cingulate, rostral anterior cingulate, insula, para-hippocampal, accumbens and hippocampus; Rt-amygdala		
		133	Deep GM	Bilateral thalamus, caudate and putamen		

Lt, left; Rt, right; AD_T_, free water-corrected axial diffusivity; FW, free water; GM, gray matter; HC, healthy controls; MD_T_, free water-corrected mean diffusivity; PD, Parkinson’s disease.

**Table 5:** Region-of-interest analyses of free-water imaging indices in the gray matter of patients with Parkinson’s disease and healthy controls according to the Braak staging.

	Braak Stage	HC		PD		*P* Value	*t*-Value	Cohen’s *d*
		Mean	SD	Mean	SD			
**MD_T_**	IV	0.56	0.039	0.60	0.036	0.016 *	−2.51	0.79
	V	0.61	0.014	0.62	0.022	0.036 °	−2.18	0.69
	VI	0.34	0.033	0.37	0.031	0.073	−1.85	0.35
**AD_T_**	IV	0.90	0.043	0.95	0.078	0.018 °	−2.48	0.78
	V	0.84	0.032	0.88	0.059	0.044 °	−2.08	0.66
	VI	0.86	0.037	0.88	0.064	0.20	−1.30	0.41
**FW**	VI	0.31	0.020	0.33	0.037	0.0059 *	−2.92	0.92
	V	0.29	0.020	0.31	0.030	0.021 °	−2.41	0.76
	VI	0.26	0.025	0.27	0.036	0.13	−1.56	0.49

AD_T_, free water-corrected axial diffusivity; DTI, diffusion tensor imaging; FW, free water; HC, healthy controls; MD_T_, free water-corrected mean diffusivity; PD, Parkinson’s disease; ROI, region of interest; SD, standard deviation. * *p* ≤ 0.025; ° 0.025 < *p* < 0.05.
